# Validation of a Brief Internet-based Self-report Measure of Maladaptive Personality and Interpersonal Schema

**DOI:** 10.1192/j.eurpsy.2023.1117

**Published:** 2023-07-19

**Authors:** H. Kim, S. Jeong, I. Hwang, W. Moon, M.-S. Shin

**Affiliations:** 1 Psychiatry, Seoul national university hospital; 2 40FY Inc., Seoul, Korea, Republic Of

## Abstract

**Introduction:**

Existing digital mental health interventions are mainly focused on the symptoms of specific mental disorders such as depression and anxiety. However, digital mental health interventions aiming enhancement of mental health in the general population are rare. Considering that the psychological discomfort of the general public is more complex and subtle, interventions focusing on maladaptive personality and interpersonal schema rather than symptoms per se can be an alternative.

**Objectives:**

To this end, concise tools for measuring the core personality and interpersonal patterns known to cause psychological discomfort among potential users of digital mental health interventions are essential. For this purpose, the Schema Scale was developed and our study aims to validate and confirm psychometric properties of the scale.

**Methods:**

This cross-sectional study was carried out between July and August 2022. Participants were 234 adults aged between 19 to 39 who completed an online survey including the Schema Scale and other 15 questionnaires. Exploratory factor analysis were conducted to construct the factorial structure model.

**Results:**

Exploratory factor analysis showed a five-factor structure with a total variance of 57%; factor 1 consisted of lack of belongingness and poor social skills, factor 2 of lack of patience hot-tempered coping style, factor 3 of maladaptive perfectionism, factor 4 of self-sacrifice and lack of self-confidence and factor 5 of items representing pessimistic and anxious mindset. Internal consistency of each factor was good(Cronbach’s alpha=0.712~0.882), and correlations with existing measures were significant.

**Conclusions:**

The five personality Schema Scale appears to be a short(total 35 items) and a valid tool for measuring five essential personality and interpersonal patterns for adults aged 20~30 years. This tool has been developed for online use and therefore has the advantage of being easily accessible. Most importantly, based on the results of the Schema Scale, the individualized digital interventions can be recommended that targets maladaptive psychological patterns.

**Disclosure of Interest:**

None Declared

